# Blood Fatty Acid Status and Clinical Outcomes in Dialysis Patients: A Systematic Review

**DOI:** 10.3390/nu10101353

**Published:** 2018-09-21

**Authors:** Ban-Hock Khor, Sreelakshmi Sankara Narayanan, Karuthan Chinna, Abdul Halim Abdul Gafor, Zulfitri Azuan Mat Daud, Pramod Khosla, Kalyana Sundram, Tilakavati Karupaiah

**Affiliations:** 1 Dietetics Program, Faculty of Health Sciences, Universiti Kebangsaan Malaysia, Kuala Lumpur 50300, Malaysia; khorbanhock@gmail.com; 2 School of Biosciences, Faculty of Health and Medical Sciences, Taylor’s University, Subang Jaya 47500, Malaysia; sreelakshmiprem07@gmail.com; 3 School of Medicine, Faculty of Health and Medical Sciences, Taylor’s University, Subang Jaya 47500, Malaysia; karuthan@gmail.com; 4 Department of Medicine, Faculty of Medicine, Universiti Kebangsaan Malaysia, Kuala Lumpur 56000, Malaysia; halimgafor@gmail.com; 5 Department of Nutrition and Dietetics, Faculty of Medicine and Health Sciences, Universiti Putra Malaysia, Serdang 43400, Malaysia; zulfitri@upm.edu.my; 6 Department of Nutrition and Food Science, Wayne State University, Detroit, MI 48202, USA; aa0987@wayne.edu; 7 Malaysian Palm Oil Council, Kelana Jaya 47301, Malaysia; kalyana@mpoc.org.my

**Keywords:** blood fatty acid, fatty acid composition, essential fatty acid, *n*-3 polyunsaturated fatty acids, dialysis, hemodialysis, peritoneal dialysis, cardiovascular disease, systematic review

## Abstract

Blood fatty acids (FAs) are derived from endogenous and dietary routes. Metabolic abnormalities from kidney dysfunction, as well as cross-cultural dietary habits, may alter the FA profile of dialysis patients (DP), leading to detrimental clinical outcomes. Therefore, we aimed to (i) summarize FA status of DP from different countries, (ii) compare blood FA composition between healthy controls and DP, and (iii) evaluate FA profile and clinical endpoints in DP. Fifty-three articles from 1980 onwards, reporting FA profile in hemodialysis and peritoneal DP, were identified from PubMed, Embase, and the Cochrane library. Studies on pediatric, predialysis chronic kidney disease, acute kidney injury, and transplant patients were excluded. Moderate to high levels of *n*-3 polyunsaturated fatty acids (PUFA) were reported in Japan, Korea, Denmark, and Sweden. Compared to healthy adults, DP had lower proportions of *n*-3 and *n*-6 PUFA, but higher proportion of monounsaturated fatty acids. Two studies reported inverse associations between *n*-3 PUFAs and risks of sudden cardiac death, while one reported eicosapentaenoic acid + docosahexaenoic acid)/arachidonic acid ratio was inversely associated with cardiovascular events. The relationship between all-cause mortality and blood FA composition in DP remained inconclusive. The current evidence highlights a critical role for essential FA in nutritional management of DP.

## 1. Introduction

Survival for most individuals with end stage kidney disease (ESKD) is by initiation of hemodialysis (HD) or peritoneal dialysis (PD). In the United States, there has been a 28% reduction in mortality rate of dialysis patients over the past 15 years but, still, the expected lifespan of incident dialysis patients remains much lower compared to their healthy counterparts [1]. Dialysis patients face a higher risk for cardiovascular disease (CVD), which accounts for 48% of overall mortality [1]. Prevention and treatment of CVD in dialysis patients remains challenging as both traditional and novel risk factors are involved in CVD pathogenesis [2]. Traditional CVD risk factors, such as obesity, hypercholesterolemia, and hypertension, are paradoxically associated with greater survival in dialysis patients [3]. Contrarily, biomarkers indicating novel or uremia-related risk factors, such as inflammation, oxidative stress, protein energy wasting, vascular calcification, anemia, and uremic toxins, have consistently been reported to be directly associated with increased CVD risk and mortality in dialysis populations [4]. 

In the general population, the circulating fatty acid (FA) profile has been suggested as a novel biomarker to monitor health-related outcomes as evidenced by recently published meta-analyses [5]. Accordingly, blood concentrations of both marine and plant *n*-3 polyunsaturated fatty acids (PUFA) have been shown to be inversely associated with total mortality and fatal cardiovascular (CV) events, whilst associations between concentrations of circulating *n*-6 PUFA and CVD outcomes remain inconclusive [5,6,7]. The role of circulating saturated fatty acids (SFA) and monounsaturated fatty acids (MUFA) on all-cause and CV mortality has also been highlighted in recent individual studies [8,9]. In contrast, the clinical implications of blood FA status in ESKD patients on dialysis have not been extensively reviewed in the literature.

It is well understood that the FA composition of blood reflects both dietary intake as well as metabolism of endogenously produced fatty acids in healthy populations [10]. Therefore, the blood FA composition provides an objective measure of dietary intake and this subject has already been extensively reviewed by Hodson et al. [11]. However, blood FA profiles are altered in the presence of chronic diseases, such as chronic respiratory diseases [12], systemic lupus erythematosus [13], cancer [14], chronic gastrointestinal disorders [15], and chronic kidney disease (CKD) [16]. Of note, related to the topic of the present review, the dialysis procedure itself affects FA metabolism [17] and alters the blood FA composition [18]. In context, the impact of dialysis on blood FA profiles and its potential implications needs to be better understood. Our objective, therefore, was to systematically review and identify studies reporting blood FA profiles in dialysis patients. In addition, we aimed to compare the blood FA profile between dialysis patients and healthy controls, and to review the evidence of blood FA status predicting clinical endpoints in dialysis patients. 

## 2. Materials and Methods

### 2.1. Data Sources, Search Strategy, and Selection

We searched the following databases through July 2018: PubMed, Embase, and Cochrane Library to identify all published original research articles reporting blood FA profile of dialysis patients. We used (“dialysis” OR “hemodialysis” OR “peritoneal dialysis” OR “end stage renal disease”) AND (“fatty acid/blood” OR “plasma fatty acid” OR “serum fatty acid” OR “phospholipid fatty acid” OR “erythrocyte fatty acid”) as search keywords. We limited the search to articles published from 1980 onwards. Wildcards such as asterisk (*) and question mark (?) were used when necessary to broaden the search results. Citations of search results from each database were exported to EndNote version X7.5.3 and duplicates were removed. Two authors (B-H.K. and S.S.N.) independently reviewed the titles and abstracts, and full texts of potential studies were retrieved for further evaluation (Table S1). In case of disagreement between the two authors, a third author (T.K.) was referred. We also performed a manual search for eligible studies by checking the reference lists of relevant original and review articles.

We included eligible studies meeting these criteria: (i) published original research articles in adult (≥18 years old) incident dialysis (HD or PD) patients; (ii) reporting at least an individual FA data of total plasma, triacylglycerol (TAG), cholesteryl ester (CE), phospholipid (PL), or erythrocyte; (iii) FA separation using a capillary column; and (iv) English language publications. We excluded (i) studies on pediatric, pre-dialysis CKD, acute kidney injury, and transplant patient groups; (ii) guidelines, case reports, conference proceedings, review articles, editorials, and letters; (iii) studies reporting FAs in free fatty acid (FFA), albumin, lipoprotein, platelet, and PL subfractions; (iv) studies reporting FA desaturation index only; and (v) duplicate publications that were published revisiting the same sampled population for further sub-analyses [19]. We checked the cross-reference to primary publication in the manuscript to identify duplicate publications. We also compared studies by author lists, study location, sample size, and baseline data reported. Duplicate publications reporting additional FA status or follow-up outcomes were included in this review, whilst duplicate publications without additional outcomes of interest were excluded.

### 2.2. Data Extraction

The baseline characteristics of included studies were extracted and tabulated. For FA data, we extracted individual FA for myristic acid (14:0), palmitic acid (PA, 16:0), palmitoleic acid (POA, 16:1*n*-7), stearic acid (SA, 18:0), oleic acid (OA, 18:1*n*-9), linoleic acid (LA, 18:2*n*-6), α-linolenic acid (ALA, 18:3*n*-3), arachidonic acid (AA 20:4*n*-6), eicosapentaenoic acid (EPA, 20:5*n*-3), adrenic acid (22:4*n*-6), docosapentaenoic acid (DPA, 22:5*n*-3), and docosahexaenoic acid (DHA, 22:6*n*-3), as well as total SFA, total MUFA, total PUFA, total *n*-3 PUFA, total *n*-6 PUFA, *n*-3 index (EPA + DHA), and *n*-6/*n*-3 PUFA ratio, whenever the data was available. For intervention studies, only baseline FA data was included, with FA data combined for two groups of subjects (intervention and control/placebo group). Furthermore, for studies comparing the FA status of pre- and post- dialysis sessions, only the FA data measured before the dialysis treatment was extracted. The FA data was extracted separately for HD and PD patients whenever available for studies involving both groups of dialysis patients. Extracted FA data was grouped according to the type of blood fraction and country. FA of total plasma and FA of total serum were grouped together, while FA of erythrocyte and FA of erythrocyte PL were grouped together [20]. The FA data was presented in relative percentage or converted to relative percentage whenever the total FA profile (sum of SFA, MUFA, and PUFA) was available. The FA value was rounded to one decimal point when presented in relative percentage and whole numbers when presented in μg/mL.

The blood *n*-3 index (EPA + DHA) status was further ranked from “very low” to “high” as previously described [20], to denote the risk of CV mortality [21]. Briefly, the relative percentage of erythrocyte EPA + DHA ≤4, >4–6, >6–8, and >8 corresponded to “very low”, “low”, “moderate”, and “high”, respectively. The categorization for total serum/plasma EPA + DHA levels were ≤2.9 (very low), >2.9–4.0 (low), >4.0–5.2 (moderate), and >5.2 (high), whereas the categorization for phospholipid EPA + DHA levels were ≤3.8 (very low), >3.8–5.7 (low), >5.7–7.6 (moderate), and >7.6 (high) [20]. 

### 2.3. Quality Assessment

Two authors (B.H.K. and S.S.N.) performed the quality assessment on studies reporting clinical endpoints using the Critical Appraisal Skills Program (CASP) Cohort Study Checklist [22]. The appraisal tool consists of three sections, which evaluate the validity and generalization of the results (Table S2).

## 3. Results

### 3.1. Characteristics of Studies Included

**Figure 1:**
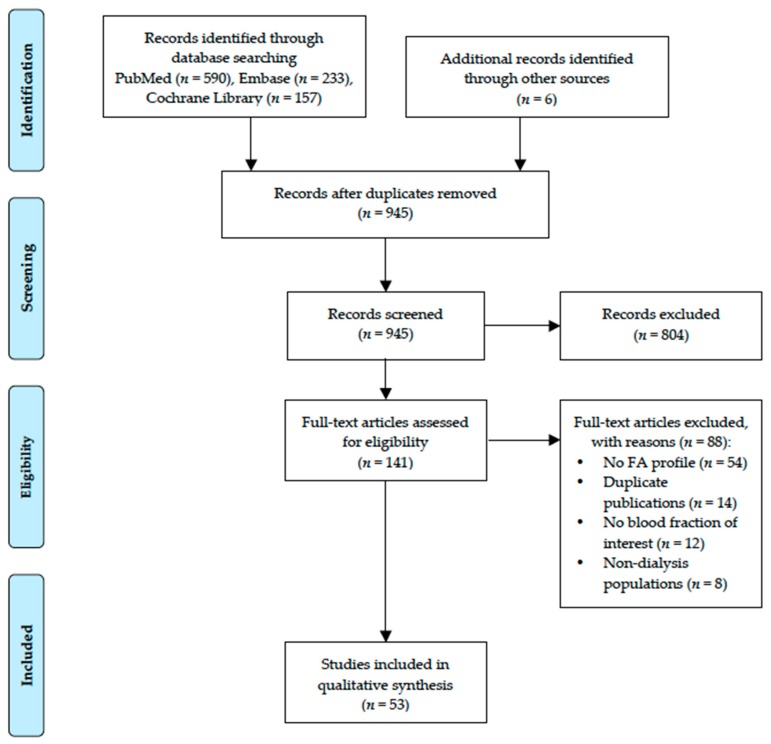
Preferred Reporting Items for Systematic Reviews and Meta-Analyses (PRISMA) study flow for literature search and study selection process. Abbreviation: FA, fatty acid.

In total, 53 studies met the inclusion criteria and were included in the present review (Figure 1). Of these studies, four were duplicate publications reporting clinical outcomes [23,24,25,26], while another one duplicate publication reported a different group of FA profile [27]. The baseline characteristics of 48 primary studies are summarized in Table 1. These were 28 cross-sectional studies, 16 interventional studies (randomized controlled trial, cross-over study or single arm intervention study), and four prospective cohort studies. Most of the studies (*n* = 34) focused only on HD patients, with some combined HD and PD patients (*n* = 8), whilst 4 studies focused only on PD patients. The dialysis modality in two studies could not be identified. The sample size ranged from 8 to 517 subjects, but only six studies enrolled more than 100 subjects. Erythrocyte FA was reported in 22 studies, whereas total plasma or serum FA composition was reported in 18 studies. Thirteen studies reported PL FA, while only five studies were reporting FA composition of TAG and/or CE.

The FA status of dialysis patients from 16 countries was identified, mainly from Japan (*n* = 8, total patients = 1135) and Korea (*n* = 8, total patients = 334), followed by the United States of America (USA) (*n* = 7, total patients = 561), Italy (*n* = 4, total patients = 159), France (*n* = 4, total patients = 84), Serbia (*n* = 3, total patients = 102), Denmark (*n* = 2, total patients = 250), Turkey (*n* = 2, total patients = 91), Poland (*n* = 2, total patients = 61), Australia (*n* = 2, total patients = 40), Sweden (*n* = 1, total patients = 222), Brazil (*n* = 1, total patients = 88), the Netherlands (*n* = 1, total patients = 32), Austria (*n* = 1, total patients = 26), South Africa (*n* = 1, total patients = 14), and Argentina (*n* = 1, total patients = 10). When the studies were categorized by continent, majority originated from Europe (*n* = 18) and Asia (*n* = 18), followed by North America (*n* = 7), South America (*n* = 2), Australia (*n* = 2) and Africa (*n* = 1).

### 3.2. Blood Fatty Acid Status

The blood FA profiles of dialysis patients are presented in Table 2. There were several variations in FA profile reported in these studies: 26 studies reported FA from SFA, MUFA, and PUFA, 14 studies reported both *n*-3 and *n*-6 PUFA only, three studies reported *n*-3 PUFA only, two studies reported SFA, MUFA, and *n*-6 PUFA only, two studies reported MUFA and PUFA only, and one study reported MUFA only. Full FA profiles were available in 12 studies only. As well, there was difference in expressing the unit of FA in terms of relative percentage (%) or absolute concentration (μg/mL). 

A distinctive FA profile with variation in proportional distribution was observed as per type of blood fraction as well as country of origin. For total serum/plasma, the most abundant FA was LA (23.2–31.5%), followed by PA and OA. However, two studies from Europe [55,67] reported greater proportion of OA (22.3–29.9%) than LA in total serum/plasma. The most abundant FA in TAG was OA (38.5–45.0%) contrasting with greater levels of LA (45.0–51.0%) in CE. The major proportions of FA in PL were PA (22.6–44.4%), LA (13.0–25.5%), and OA (13.0–18.0%). On the other hand, the highest concentration of FA in erythrocyte was PA (21.5–30.3%), followed by SA, OA, LA, and AA. However, a study from USA [40] reported AA (17.7%) as the most abundant FA in erythrocytes.

For *n*-3 index status, moderate to high levels of EPA + DHA in total serum/plasma were reported in studies from Japan, ranging from 3.1 to 6.4%. Contrarily, dialysis patients from Turkey, North America, and South America exhibited low to very low levels of total serum/plasma EPA + DHA (1.6–2.2%). Most studies did not report *n*-3 PUFA in TAG and CE. Only Friedman et al. [41] reported the median value of zero for both EPA + DHA in non-polar blood fraction (TAG + CE), while Yoshimoto-Furuie et al. [73] reported 4.5% and 5.0% for EPA + DHA levels in CE and TAG respectively, in Japanese dialysis patients. The EPA + DHA levels in PL reported in studies from Japan (6.8%) and Sweden (6.5%) were considered moderate, whereas low levels of PL EPA + DHA were observed in dialysis patients from Denmark (5.5–5.7%) and Australia (5.2%). Very low levels of EPA + DHA in PL were reported in studies from Serbia (3.0–3.3%) and USA (~2.8%). High levels of erythrocyte EPA + DHA were reported in studies from Japan (9.7%) and Korea (>8%), whereas studies from Italy and France reported low and moderate levels of EPA + DHA in erythrocyte (4.8–6.8%). Very low and low levels of erythrocyte EPA + DHA were observed in studies from USA (3.4–5.0%), Serbia (2.2–4.5%), and South Africa (3.9%).

### 3.3. Blood FA Status of Dialysis Patients Compared to Healthy Controls

Twenty-two studies compared the blood FA status of dialysis patients against healthy controls (Table 3). Most studies did not report significantly different proportions of SFA in all blood fractions. For total serum/plasma, higher levels of OA and MUFA, in parallel with lower levels of LA, AA, EPA, DHA, total *n*-3 PUFA, and total PUFA, were consistently reported. Only one study compared the FA of TAG and CE in dialysis patients to healthy controls, and this study observed lower levels of LA in dialysis patients compared to healthy controls in both TAG (0.8% vs. 4.0%) and CE (2.9% vs. 14.0%) [68]. Similar trends of elevated levels of OA and MUFA concomitant with lower total *n*-6 PUFA, EPA, DHA, and total *n*-3 PUFA levels, were reported for PL. Both similar and lower proportions of PL LA and AA levels in dialysis patients compared to healthy controls were reported. For erythrocyte FAs, lower levels of POA, LA, ALA, DHA, and total PUFA in dialysis patients were observed. Differences in erythrocyte OA, total MUFA, AA, total *n*-6 PUFA, EPA, and DPA levels in dialysis patients compared to healthy controls were not consistently reported. Fifteen studies that included data on mean dialysis vintage were further stratified into either dialysis vintage below or ≥72 months (Table S3). Lower proportions of total plasma/serum *n*-3 PUFA and PL *n*-6 PUFA were reported with mean dialysis vintage below 72 months, but not ≥72 months. By contrast, erythrocyte FA comparisons were similar, irrespective of dialysis vintage period.

### 3.4. Blood FA Predicting Clinical Endpoints

Six prospective cohort studies and one retrospective study [26] reported association between blood FA status and clinical endpoints such as CV events, all-cause mortality, and sudden cardiac death (Table 4). Six studies focused on HD patients only, while one study included both HD and PD patients [47].

Shoji et al. [63] investigated the relationship between total serum FA and CV events in HD patients and reported that individual AA, EPA, and DHA were not significantly associated with risk of CV events (data not presented in the article). However, a lower ratio of (EPA+DHA)/AA (0.63–0.83) was found to be associated with a higher hazard ratio (HR) of CV events (HR: 1.92; 95% confidence interval (CI): 1.25–2.95) [63]. On the other hand, Friedman et al. [23,24] examined the associations between risks of sudden cardiac death and FA of total serum and PL during the first year of HD initiation. They reported that higher levels of PL total long-chain FAs (4.51–15.11%) were associated with a lower odds ratio (OR) of sudden cardiac death (OR: 0.20; 95% CI: 0.08–0.51) [24]. In addition, both total serum and PL DPA were inversely associated with lower odds of sudden cardiac death, while every 0.1% increase in total serum SFA was associated with 1% increased odds of sudden cardiac (OR: 1.01, 95% CI: 1.00–1.02, *p* = 0.0258) [23]. However, it is important to note that the lower limit of the 95% CI is 1.00. The *p* value being less than 0.05 could be due to the sample size effect (*n* = 400). Therefore, the clinical relevance of this analysis is uncertain.

In regard to the risk of all-cause mortality, a retrospective study reported that all-cause mortality risks in HD patients with erythrocyte *n*-3 index below median (4.69%) were not significantly higher (HR: 2.48; 95% CI: 0.88–6.95, *p* = 0.085) compared to those with erythrocyte *n*-3 index above median [26]. Shoji et al. [63] also reported no significant association between overall mortality and individual levels of AA, EPA, DHA, and (EPA+DHA)/AA ratio. Similarly, Huang et al. [47] reported that PL ALA and long chain *n*-3 PUFAs were not associated with lower risk of all-cause mortality in dialysis (HD and PD) patients. Instead, they reported every 1% increase in PL LA was associated with 11% lower risk of all-cause mortality (HR: 0.89; 95% CI: 0.79–0.99), while every 0.1% increase in PL mead acid (20:3*n*-9) was associated with 33% increased risk of all-cause mortality (HR: 1.33; 95% CI: 1.17–1.52). In contrast to these observations, Hamazaki et al. [45] reported that higher levels of erythrocyte DHA (>8.1%) were significantly associated with reduced risk of all-cause mortality (HR: 0.43; 95% CI: 0.21–0.88) in HD patients during a 5-year follow-up study, and similar findings were also reported when the follow-up was extended for up to 10 year (HR: 0.45; 95% CI: 0.31–0.91) [25]. This study also reported that higher erythrocyte OA proportions were associated with lower all-cause mortality in HD patients (HR: 0.46; 95% CI: 0.25–0.84) [25].

## 4. Discussion

To our knowledge, this is the first systematic review to examine the circulating FA profile in dialysis patients and its potential clinical implications. Analysis of FA composition of various biological specimens as biomarkers of dietary intake in population-based studies has been reported in the literature, relating to adipose tissue, plasma, erythrocytes, and platelets [11]. In the present review, we included total plasma/serum, TAG, CE, PL, and erythrocytes. Other blood fractions were excluded, due to our finding that only few studies reported these parameters. There were four studies reporting FFA published before the year 2002, essentially to assess the effect of heparin on FA profile in FFA. It should be noted that additionally blood analyses were likely performed for both fasting (*n* = 28) and non-fasting (*n* = 4) samples of patients from the included studies. Therefore, any determination of circulating FFA may not ideally differentiate between non-esterified FAs from storage adipose tissue or FFAs from postprandial release by lipolytic action on chylomicron TAGs [11]. Although the FA compositions are typical and specific to each biological specimen, changes in FA profile in response to dietary manipulation have been demonstrated in intervention trials [10]. Studies in dialysis patients have shown that supplementation of marine *n*-3 PUFA resulted in incorporation of *n*-3 PUFA in total plasma [33,35,75], TAG [36], PL [66,74], and erythrocytes [32,75]. Apart from being a biomarker of dietary intake, circulating FAs also have major physiological roles. For instance, PUFAs in the PL membrane are involved in maintaining the fluidity and structural integrity of cell membranes, as well as serving as the direct precursors for eicosanoid biosynthesis [76].

In the present review, we observed the geographical disparities in blood *n*-3 index levels in dialysis patients, which was consistent with the findings from a global survey on circulating *n*-3 PUFA status of healthy adults [20]. Healthy adults from countries on the Sea of Japan (Japan and South Korea) and Scandinavia (Denmark and Sweden) had high blood levels of EPA + DHA, while low to very low levels of EPA + DHA were observed in healthy adults from North and South America, Africa, and Serbia [20]. This is likely due to the dietary diversity related to food choices as well as fats and oils consumption across nations [77]. We have previously shown in a meta-analysis that *n*-3 PUFA supplementations were able to reduce C-reactive protein (CRP) in HD patients [78]. Therefore, the regional variations of blood *n*-3 PUFA status in dialysis patients could be a plausible explanation for the differences in CRP levels reported in the Dialysis Outcomes and Practice Patterns Study, where Japanese HD patients exhibited lower CRP levels (1.0 mg/L) than their counterparts from other countries (6.0 mg/L) [79].

In comparison to healthy adults, dialysis patients exhibit lower concentrations of blood essential FAs (LA and ALA) and their respective metabolic derivatives (AA, EPA, and DHA). The gradual loss of renal residual function may alter plasma FA profiles as differences in plasma PUFA levels were reported in pre-dialysis patients at stage 5 CKD, but not stage 3–4 CKD patients [16]. One study which compared the plasma FA composition between pre-dialysis CKD and HD patients also observed that HD patients had lower plasma *n*-3 PUFA [53]. Four studies investigated the effects of HD treatment on FA composition of total plasma, PL, and erythrocyte [58,59,67,80]. Surprisingly, the proportion of essential FAs (LA and ALA) remained unchanged after the 4 h HD treatment. However, one study reported reductions in plasma DPA and DHA [59]. Contrarily, an acute rise in plasma AA, EPA, and DHA, as well as PL AA, adrenic acid, and DPA, were reported by Friedman et al. [81] and Peuchant et al. [58], respectively. Therefore, we postulate that the stage of kidney disease rather than the HD treatment is involved in modification of blood FA composition.

Possible mechanisms that may lead to an altered FA profile in CKD patients include (i) Altered FA metabolic pathways, such as fatty acid oxidation and PUFA biosynthesis, were observed in a CKD rat model, attributed to reduced expression of key enzymes related to FA metabolism [81]. (ii) Progressive decline in renal function leads to reduced clearance of pro-inflammatory cytokines and elevations of oxidative stress and inflammatory biomarkers in the uremic state, which have been documented in CKD patients even before initiation of dialysis [82]. An increase in oxidative stress and inflammation could induce membrane lipid peroxidation, and PUFAs containing double bonds are more susceptible to attack by free radicals [83]. (iii) Uremic anorexia in CKD patients causes poor oral intake [84] and, therefore, suboptimal consumption of dietary PUFA [85], which subsequently leads to deficiency in essential FAs. (iv) Inadequate fish consumption as plasma and erythrocyte *n*-3 PUFA levels were reported as reflections of the frequency of fish consumption in HD patients [40]. (v) Endogenous synthesis of EPA and DHA occurs from the chain elongation of ALA, but their conversion in humans is considered relatively inefficient [86] and may be also greatly hindered in the uremic milieu [40].

Dialysis patients also have higher proportion of circulating OA and total MUFA compared to healthy adults, which may be linked to detrimental outcomes. A recent prospective cohort study in non-dialysis patients (*n* = 3259) showed that OA in erythrocytes was directly associated with markers of oxidative stress (oxidized low-density lipoprotein), inflammation (interleukin-6), and endothelial activation (intracellular adhesion molecule 1, fibrinogen, and galectin-3), as well as all-cause and CV mortality over a median follow-up of 10 years [8]. In fact, patients with coronary artery disease were also presented with higher erythrocyte OA and total MUFA [87]. In another study on pre-dialysis patients, an individual *n*-9 MUFA, namely nervonic acid (24:1*n*-9) was associated with increased all-cause mortality [16]. Specific to dialysis patients, a cross-sectional study in HD patients by Son et al. [65] showed that erythrocyte OA and total MUFA were significantly associated with vascular calcification score estimated by plain radiographs, which had been previously shown to be an independent predictor of CV risk or mortality in HD patients [88]. Unexpectedly, Terashima et al. [25] reported inverse associations between erythrocyte OA and all-cause mortality in HD patients, which contrasted with other studies [8,65,87]. The implication of this finding is uncertain. We observed higher total serum OA proportions in studies originating from Italy [67] and Poland [55], which may be related to higher dietary MUFA consumptions in these populations [89]. However, the increase in blood 18:1*n*-9 levels may also be beyond dietary origins, as this FA can be synthesized endogenously [10]. One may speculate that the increase in proportions of circulating 18:1*n*-9 and total MUFA is a compensatory metabolism to reduced circulating levels of *n*-3 and *n*-6 PUFA [15]. It is hypothesized that more MUFAs are produced to substitute *n*-3 and *n*-6 PUFA as an attempt to maintain membrane fluidity during the state of essential FA deficiency [15]. In fact, it has been demonstrated that the supplementation of *n*-3 PUFA in dialysis patients resulted in reduced erythrocyte content of total SFA, OA, and total MUFA, alongside increases in erythrocyte EPA, DHA, and total *n*-3 PUFA levels [30]. It is also worth noting that PD patients have higher erythrocyte MUFA, POA, and OA, than HD patients [28,43,48]. A higher carbohydrate load from the peritoneal glucose dialysate is likely to promote de novo synthesis of MUFA of *n*-7 and *n*-9 series [90].

The current evidence suggests that circulating *n*-3 PUFA is associated with lower risk of CV events and mortality in HD patients, which is in agreement with the findings in healthy populations [7]. A randomized controlled trial (*n* = 206) in HD patients demonstrated that *n*-3 PUFA supplementation reduced the number of myocardial infarctions as a secondary outcome [66]. The putative cardioprotective mechanisms of *n*-3 PUFA include modification of cell membranes, attenuation of ion channels, regulation of pro-inflammatory gene expression, and production of eicosanoids [91]. However, the evidence on associations between *n*-3 PUFA and all-cause mortality remains inconclusive, as different blood fractions investigated may lead to finding discrepancies such as significant associations with all-cause mortality that were observed for erythrocyte [25,45], but not with total serum or PL [47,63]. Contrary to expectations, Huang et al. [47] observed that PL LA, instead of *n*-3 PUFA, was inversely associated with all-cause mortality in dialysis patients. The *n*-6 PUFA is generally perceived as the culprit of chronic inflammatory disease by being the precursor of pro-inflammatory eicosanoids. However, the role of *n*-6 PUFA in moderating inflammation response has become the subject of recent debate, as the *n*-6 PUFA also gives rise to eicosanoids involved in resolution of inflammation [92]. Recent evidence from epidemiological studies [93,94] and a meta-analysis of randomized controlled trial [95] showed that LA did not actually increase the concentrations of inflammatory markers. In addition, Huang et al. [47] also reported that PL mead acid, an indicator of essential FA deficiency [96], was associated with increased all-cause mortality in dialysis patients. Therefore, greater levels of circulating LA may confer beneficial effects to dialysis patients, who are prone to essential FA deficiency.

Our review has several limitations. Firstly, we included only publications in the English language, which may lead to exclusion of FA data from scientific publications in other languages. Second, we were unable to convert the FA data of some studies into similar units for comparison if these studies lack reporting total FA. Third, sample sizes of most studies were relatively small (<100 patients), therefore, the FA data may not be truly representative of that population. Fourthly, we were not able to conduct a meta-analysis to examine the association between circulating FA and clinical endpoints in dialysis patients due to the heterogeneity of outcomes and nature of FA reported in each study. Lastly, the effects of type of dialyzer and HD treatment on FA profiles could not be properly validated due to limited studies reported in the literature. Despite these limitations, our review provides an extensive overview on the blood FA profile of dialysis patients from various countries, FA pattern modifications in dialysis patients, as well as clinical implications related to it.

## 5. Conclusions

Dialysis patients having altered blood FA profiles present with increased MUFA and reduced PUFA levels. The available evidence suggests that low levels of circulating PUFAs were associated with increased risks of CV events and mortality in dialysis patients. Therefore, it is necessary to establish a reference range for blood PUFA profile in these patients, which can be used as a biomarker for risk assessment. As the FA composition in blood is influenced by dietary intakes, medical nutrition therapy for dialysis patients should also include dietary modifications that ensure adequate consumptions of essential FA, particularly *n*-3 PUFA. Most studies available have focused on HD patients and only a few included PD patients, suggesting that more research related to blood FA profiles in PD patients is warranted.

## Figures and Tables

**Table 1:** Summary of studies included in the review.

Author (year)	Country	*n*	Mean Age (year)	Gender (M/F)	Dialysis	Dialysis Vintage (month)	Study Type	Blood Fraction	Instrumentation
An (2009) [28]	Korea	29	59.5	15/14	HD, PD	43.6	CS	Erythrocyte	GC
An (2011) [29]	Korea	73	57.3	44/29	HD, PD	72.3	CS	Erythrocyte	GC
An (2012) [30]	Korea	14	52.1	7/7	PD	46.9	INT	Erythrocyte	GC
An (2012) [31]	Korea	43	57.4	20/23	HD, PD	46.5	INT	Erythrocyte	GC
Begum (2004) [32]	USA	22	55.8	13/9	HD	63.7	INT	Erythrocyte	GLC
Dasgupta (1990) [18]	USA	9	46.0	3/6	HD	72.0	CS	Total plasma	GC-MS
de Fijter (1995) [33]	NL	32	N/A	N/A	N/A	N/A	INT	PL	GC-FID
de Gomez Dumm (2001) [34]	Argentina	10	33.3	6/4	HD	60	PC	Total plasma	GLC-FID
de Mattos (2017) [35]	Brazil	88	52.0	57/31	HD	54.4	INT	Total serum	GC
Delarue (2008) [36]	France	8	62.0	6/2	HD	≥ 6	INT	TAG	GC
Delmas-Beauvieux (1995) [37]	France	40	58.1	19/21	HD	≥ 6	CS	Erythrocyte	GC
Dessi (2014) [38]	Italy	99	69.3	59/40	HD	65.8	CS	PL, Erythrocyte	GC-MS
Esaki (2017) [39]	Japan	10	74.7	7/3	HD	100.8	INT	Total serum	N/A
Friedman (2006) [26,40]	USA	75	53.8	48/27	HD	N/A	CS	Total plasma, erythrocyte	GC-FID
Friedman (2012) [23,24,41]	USA	400	66.4	232/ 168	HD	N/A	CS	Total serum, PL, TAG & CE	GC-FID
Friedman (2016) [42]	USA	20	55.0	11/9	HD	96.0	CS	PL	GC
Girelli (1992) [43]	Italy	32	61.9	16/16	HD, PD	42.0	CS	Erythrocyte	GC
Hamazaki (1984) [44]	Japan	12	N/A	3/9	HD	31.0	INT	Total plasma	GC
Hamazaki (2011) [25,45]	Japan	176	64.1	96/80	HD	92.4	PC	Erythrocyte	GC
Holler (1995) [46]	Austria	26	48.2	14/12	PD	N/A	CS	Total serum	GC
Huang (2012) [27,47]	Sweden	222	57.0	135/87	HD, PD	12.0	PC	PL	GLC
Kim (2013) [48]	Korea	61	56.0	44/17	HD, PD	48.1	CS	Erythrocyte	GC
Koorts (2002) [49]	S. Africa	14	37.3	9/5	HD	69.9	CS	Erythrocyte	GLC-FID
Lee (2015) [50]	Korea	15	62.1	5/10	HD	≥ 6	INT	Erythrocyte	GC
Madsen (2011) [51]	Denmark	44	63	29/15	HD	30.0	CS	PL	GC-FID
Marangoni (1992) [52]	Italy	18	48.7	10/8	HD	≥ 6	CS	TAG, CE, PL	GLC
Nakamura (2008) [53]	Japan	17	57.0	N/A	HD	N/A	CS	Total plasma	GC
Oh (2012) [54]	Korea	68	56.4	27/41	HD, PD	49.0	CS	Erythrocyte	GC
Pazda (2017) [55]	Poland	28	50.7	15/13	PD	N/A	CS	Total serum	GC-FID
Peck (1996) [56]	USA	25	49.8	13/12	HD	N/A	INT	Total plasma	GC
Perunicic-Pekovic (2007) [57]	Serbia	35	N/A	N/A	HD	N/A	INT	Erythrocyte	GLC
Peuchant (1988) [58]	France	22	N/A	N/A	HD	78.0	CS	Erythrocyte	GC-FID
Peuchant (1994) [59]	France	14	51.0	5/9	HD	96.0	CS	Total plasma, erythrocyte	GLC
Ristic (2006) [60]	Serbia	37	52.0	21/16	HD	72.0	CS	PL, erythrocyte	GC
Ristic-Medic (2014) [61]	Serbia	30	55.0	18/12	HD	57.1	INT	PL	GC
Sertoglu (2014) [62]	Turkey	40	58.0	21/19	HD	N/A	CS	Total plasma, erythrocyte	GC-FID
Shoji (2013) [63]	Japan	517	61.0	325/ 192	HD	110.4	PC	Total serum	GC
Sikorska-Wisiewska (2017) [64]	Poland	33	55.8	18/15	HD, PD	12.2	CS	Total serum	GC-EI-MS
Son (2012) [65]	Korea	31	56.2	10/21	HD	46.1	CS	Erythrocyte	GC
Svensson (2006) [66]	Denmark	206	67.0	133/73	HD	44.0	INT	PL	GC-FID
Taccone-Galluci (1989) [67]	Italy	10	N/A	6/4	HD	27.0	CS	Total serum	GC
Talwalker (1980) [68]	USA	10	49.0	10/0	N/A	N/A	CS	TAG & CE, PL	GLC
Tsuzuki (2000) [69]	Japan	20	55.6	11/9	HD	80.4	CS	Erythrocyte	GC-MS
Umemoto (2016) [70]	Japan	367	66.0	237/130	HD	109.2	CS	Total serum	GC
Westhuyzen (2003) [71]	Australia	12	69.2	7/5	HD	N/A	INT	Erythrocyte	GC-FID
Yerlikaya (2011) [72]	Turkey	51	47.8	21/30	PD	65.4	CS	Total plasma	GC-MS
Yoshimoto-Furuie (1999) [73]	Japan	16	52.7	6/10	HD	62.4	INT	TAG, CE, PL	GC
Zabel (2010) [74]	Australia	28	61.0	14/14	HD	19.5	INT	PL	GC

Abbreviations: CE, cholesteryl ester; CS, cross-sectional; F, female; FID, flame ionized detector; GC, gas chromatography; GC-EI-MS, gas chromatography-electron ionization-mass spectrometry; GC-MS, gas chromatography-mass spectrometry; GLC, gas liquid chromatography; HD, hemodialysis; INT, intervention; M, male; N/A, not available; NL, the Netherlands; PC, prospective cohort; PD, peritoneal dialysis; PL, phospholipid; S. Africa, South Africa; TAG, triacylglycerol; USA, United States of America.

**Table 2:** Blood fatty acid profiles (relative percentage) of dialysis patients.

Author (year)	Country	14:0	16:0	18:0	Total SFA	16:1*n*-7	18:1*n*-9	Total MUFA	18:2*n*-6	20:4*n*-6	22:4*n*-6	Total *n*-6 PUFA	18:3*n*-3	20:5*n*-3	22:5*n*-3	22:6*n*-3	*n*-3 index	Total *n*-3 PUFA	Total PUFA	*n*-6/*n*-3
**Total Serum/Plasma**																				
	**Asia**																			
Hamazaki (1984) [44]	Japan		22.7	5.0		3.5	22.6		31.5	3.8			1.1	1.2		1.9				
Nakamura (2008) [53]	Japan								30.1	5.2	0.1		0.8	2.3	0.7	4.1				
Esaki (2017) [39]	Japan		21.9	5.9			25.7		27.4	5.8			1.0	1.1		2.9				
Shoji (2013) ^a,b^ [63]	Japan									***139***				***53***		***100***				
Umemoto (2016) ^a,b^ [70]	Japan									***149***		***173***		***60***		***100***		***165***		
Yerlikaya (2011) [72]	Turkey	0.7	21.6	8.1	34.5	1.5	9.2	27.5	23.7	4.6		28.6		0.7		0.9		2.6	38.0	19.5
Sertoglu (2014) ^a^ [62]	Turkey	***48***	***308***	***104***		***26***	***357***		***495***	***115***		***634***		***6***		***15***		***20***		
	***Europe***																			
Taccone-Galluci (1989) [67]	Italy	1.3	21.6	8.0		2.1	29.9		20.6	7.3	0.6		0.6		0.9	2.6				
Peuchant (1994) [59]	France	1.3	26.0	11.3			22.3		24.2	7.7	0.6				0.9	2.6				
Holler (1996) [46]	Austria									6.1				0.4						
Pazda (2017) [55]	Poland	1.0	23.4	7.1	32.5	2.8	29.1	32.7	23.2	5.4	0.1	29.9	0.3	0.8	0.4	1.5		3.1		
Sikorska-Wisiewska (2017) [64]	Poland						26.3			3.8		4.0	0.2	0.5		1.2		3.4		
							28.4			3.7		3.9	0.2	0.6		1.1		3.6		
	***North America***																			
Dasgupta (1990) [18]	USA	2.5	21.9	14.3		4.7	15.4		26.6	6.0			0.2		0.7	2.1				
Peck (1996) [56]	USA						20.0		28.0	5.7			0.6	0.5						
Friedman (2006) [40]	USA	0.6	19.8	7.6	28.4	1.4	23.6	27.3	26.7	8.4	0.3		0.7	0.4	0.4	1.3	1.7		40.4	
Friedman (2012) ^b^ [41]	USA		20.2	6.8	28.0	2.3	23.9	28.2	28.3	7.5			0.5	0.3	0.4	1.3			40.9	
	***South America***																			
de Gomez Dumm (2001) [34]	Argentina		19.4	6.4		3.1	24.3		31.9	7.5	0.9			0.6	0.6	1.4				
de Mattos (2017) [35]	Brazil									5.6			0.7	0.6		0.6				
**Triacylglycerol**																				
	***Asia***																			
Yoshimoto-Furuie (1999) [73]	Japan								23.6	1.6			2.1	1.2		3.8				
	***Europe***																			
Marangoni (1992) [52]	Italy		31.0	5.0		4.0	45.0		12.0	1.0										
Delarue (2008) ^a^ [36]	France													***8***		***10***				
	***North America***																			
Talwalker (1980) [68]	USA	3.4	38.9	7.1		3.8	38.8		0.8	0.9										
**Cholesteryl Esters**																				
	***Asia***																			
Yoshimoto-Furuie (1999) [73]	Japan								51.0	6.0			0.6	2.9		1.7				
	***Europe***																			
Marangoni (1992) [52]	Italy		15.0	2.0		5.0	26.0		45.0	6.0										
	***North America***																			
Talwalker (1980) [68]	USA	3.7	30.6	4.8		6.3	32.2		2.9	5.1										
**Triacylglycerol and Cholesteryl Esters**																				
Friedman (2012) ^b^ [41]	USA		17.8	4.4	22.4	2.4	28.1	32.8	33.3	5.1			0	0	0	0			39.3	
**Phospholipids**																				
	***Asia***																			
Yoshimoto-Furuie (1999) [73]	Japan								23.1	9.1	0.3		0.4	3.1	1.1	7.6				
	***Australia***																			
Zabel (2010) [74]	Australia									10.3				1.1		4.1				
	***Europe***																			
Marangoni (1992) [52]	Italy		37.0	15.0		1.0	13.0		13.0	8.0										
Dessi (2014) ^a^ [38]	Italy								***408***	***133***			***5***	***9***		***49***				
de Fijter (1995) [33]	NL													4.3						
Svensson (2006) [66]	Denmark													1.5		4.0				
Madsen (2011) [51]	Denmark									9.7				1.6	1.1	4.1				
Ristic (2006) ^b^ [60]	Serbia		28.1	15.7	43.8	0.4	13.1	13.5	25.5	11.1	0.4	39.0		0.3	0.5	3.0		3.8		9.6
Ristic-Medic (2014) [61]	Serbia		25.3	16.4	41.8	0.4	13.8	14.6	24.5	11.6	0.6	39.3	0.1	0.2	0.5	2.8		3.5	42.7	11.3
Huang (2012) [27,47]	Sweden		30.4	14.5		0.5	13.7		19.7	9.2			0.3	1.6	1.2	4.9			39.9	
	***North America***																			
Talwalker (1980) [68]	USA	2.9	44.4	21.7		3.0	18.0		1.8	1.2										
Friedman (2012) ^b^ [41]	USA		22.6	17.6	40.9	2.4	15.6	19.1	18.7	10.5			0.3	0.3	0.8	2.8			36.9	
Friedman (2016) [42]	USA								19.2	13.5				0.4		2.4				
**Erythrocytes**																				
	***Asia***																			
Tsuzuki (2000) [69]	Japan				54.0			19.2	8.5		1.6				1.2	4.9			26.8	
Hamazaki (2011) [45]	Japan		26.8	15.0		0.4	13.4		9.1	11.6				2.0	2.5	7.7				2.0
An (2009) [28]	Korea	0.2	22.6	16.4	39.2	0.6	12.8	14.0	11.9	14.7	1.5	29.8	0.3	3.1	3.1	10.2	13.3	16.7	46.3	1.9
		0.3	23.2	15.8	39.4	0.9	14.5	16.0	10.5	14.7	1.5	28.4	0.3	3.0	2.7	9.8	12.8	15.9	44.3	1.9
An (2011) [29]	Korea									14.7		29.8	0.3	3.1	3.1	10.2		16.7		
										14.7		28.4	0.3	3.0	2.7	9.8		15.9		
An (2012) [30]	Korea	0.7	23.5	11.5	35.7	1.2	17.1	18.5	18.6	12.0		33.5	0.7	1.7		7.1	8.9	11.1	44.7	3.1
An (2012) [31]	Korea	0.6	28.0	17.2	46.0	2.1	16.8	19.5	13.0	10.2		26.0	0.5	1.3		2.9	4.0	5.4	31.5	6.2
Oh (2012) [54]	Korea	0.7	23.8	12.1	36.8	1.4	16.9	18.7	18.1	11.1		31.9	0.6	2.0	1.5	6.6	8.6	10.7	42.7	3.4
Son (2012) [65]	Korea	0.6	23.3	12.2	36.3	1.0	16.2	17.6	18.5	11.4	1.2	32.6	0.5	2.1	1.7	7.3	9.4	11.7	44.3	
Kim (2013) [48]	Korea						16.1	17.6												
							17.7	19.7												
Lee (2015) [50]	Korea	0.5	25.6	19.4	46.0	0.7	15.9	17.6	9.8	10.6		24.6	0.3	1.4		6.7	8.1	10.6	35.2	2.8
Sertoglu (2014) ^a^ [62]	Turkey	***33***	***22***	***51***		***8***	***30***		***35***	***42***		***83***		***3***		***5***		***6***		
	***Australia***																			
Westhuyzen (2003) [71]	Australia		22.8	16.9	43.8		15.5	19.5	8.6	16.7	3.3			0.8		7.3			36.7	
	***Europe***																			
Girelli (1992) [43]	Italy		21.5	16.7	44.4		15.5	16.0	8.4	23.5						6.8			39.3	
			21.7	17.1	46.4		17.4	17.9	8.4	19.8						6.4			35.4	
Dessi (2014) ^a^ [38]	Italy								***117***	***145***			***0.2***	***3***		***45***				
Peuchant (1988) [58]	France	0.8	29.4	23.0			13.4		7.9	11.7	2.1			0.5		3.2				
Peuchant (1994) [59]	France	0.8	25.7	22.6			13.4		9.5	13.8	2.7				2.7	4.1				
Delmas-Beauvieux (1995) [37]	France								12.5	11.9	2.3				1.6	4.8				
Ristic (2006) [60]	Serbia		21.6	19.3	40.9		17.9	17.9	14.8	15.3	3.5	34.9		0.2	1.2	4.3		6.0		5.9
Perunicic-Pekovic (2007) [57]	Serbia									7.4				0.2	0.6	2.0				
	***Africa***																			
Koorts (2002) [49]	South Africa	0.3	22.3	17.4	45.9	0.2	13.3	16.9	10.4	14.8	3.9	31.7	0.2	0.2	1.4	3.7		5.6	37.2	5.8
	***North America***																			
Begum (2004) [32]	USA		30.3	24.5			23.4		9.0	6.9	1.8	18.9	0.2	0.1	0.6	1.8		2.7		
Friedman (2006) [26,40]	USA	0.1	15.0	15.7	31.2	0.2	13.9	11.2	9.4	17.7	5.2		0.03	0.3	2.4	4.7	5.0		42.9	

Data highlighted in grey represents FA profile of PD patients alone or combining of HD and PD patients, ^a^ Data is in μg/mL (bolded and italicized), ^b^ Data is presented as median. Abbreviations: MUFA, monounsaturated fatty acid; *n*-3 PUFA, omega-3 polyunsaturated fatty acid; *n*-6 PUFA, omega-6 polyunsaturated fatty acid; NL, the Netherlands; PUFA, polyunsaturated fatty acid; SFA, saturated fatty acid; USA, United States of America. Fatty acid abbreviations: 14:0, myristic acid; 16:0, palmitic acid; 16:1*n*-7, palmitoleic acid; 18:0, stearic acid; 18:1*n*-9, oleic acid; 18:2*n*-6, linoleic acid; 18:3*n*-3, α-linolenic acid; 20:4*n*-6, arachidonic acid; 20:5*n*-3, eicosapentaenoic acid; 22:4*n*-6, adrenic acid; 22:5*n*-3, docosapentaenoic acid; 22:6*n*-3, docosahexaenoic acid.

**Table 3:** Comparison of FA status of dialysis patients to healthy controls.

	Total Serum/Plasma	TAG/CE [68]	PL	Erythrocyte
SFA				
14:0	↔ [18,40,55,59,62,72]	↔	↔ [68]	↔ [49,54,58,59,62], ↓ [28,40]
16:0	↔ [18,40,55,59,62,72], ↑ [34]	↔	↔ [60,68]	↔ [28,49,54,58,59,60,62], ↓ [40,71]
18:0	↔ [18,34,40,55,62,72], ↓ [59]	↔	↔ [60,68]	↔ [28,40,49,58,60,62], ↑ [59,71], ↓ [54]
Total SFA	↔ [40,55], ↑ [72]	↔	↔ [60]	↔ [28,49,60,71], ↓ [40,54], ↑ [69]
MUFA				
16:1*n*-7	↔ [18,40,55,62,72], ↑ [34]	↔	↔ [60,68]	↓ [28,40,62], ↔ [49,54]
18:1*n*-9	↑ [18,34,40,55,56,59,64], ↔ [62,72]	↔	↑ [60], ↔ [68]	↔ [40,58,60,62,71], ↑ [28,49,54], ↓ [59]
Total MUFA	↑ [40,55,72]	↔	↑ [60]	↔ [40,60,69,71], ↑ [49,54], ↓ [28]
*n*-6 PUFA				
18:2*n*-6	↓ [18,34,40,55,59,72], ↔ [56,62]	↓	↔ [42,60], ↓ [38,68]	↓ [38,40,62,69], ↔ [49,58,59,60,71], ↑ [28,54]
20:4*n*-6	↓ [18,34,56,59,64,72], ↔ [40,46,55,62], ↑ [51,63]	↔	↔ [60,68], ↓ [38], ↑ [42]	↔ [49,54,58,60,62,71], ↑ [28,29,40,59], ↓ [38,57]
22:4*n*-6	↔ [40,55,59], ↓ [34]		↔ [60]	↔ [49,58,59,60,71], ↓ [28,69], ↑ [40]
Total *n*-6 PUFA	↔ [62,72], ↓ [55,64]		↓ [60]	↑ [28,29,54], ↔ [60,62], ↓ [49]
*n*-3 PUFA				
18:3*n*-3	↔ [40,56], ↓ [18,55,64]		↔ [38]	↓ [28,29,38,40], ↔ [49], ↑ [54]
20:5*n*-3	↓ [34,46,51,56,64], ↔ [40,55,62,72], ↑ [63]		↓ [38,42,60]	↔ [28,29,38,40,54,58,62], ↓ [49,57,60,71]
22:5*n*-3	↔ [18,40,59], ↓ [34], ↑ [55]		↔ [60]	↔ [28,29,49,54,57,60], ↓ [69], ↑ [40,59]
22:6*n*-3	↓ [34,40,51,64,72], ↔ [18,59,62], ↑ [55,63]		↔ [38,42], ↓ [60]	↓ [38,54,57,60,62], ↔ [28,29,49,59,69,71], ↑ [40,58]
*n*-3 Index	↓ [40]		↔ [38]	↔ [28,38], ↑ [40], ↓ [54]
Total *n*-3 PUFA	↓ [64,72], ↔ [55,62]		↓ [60]	↔ [28,29,49,62], ↓ [54,60]
Total PUFA	↓ [40], ↔ [72]			↓ [49,69], ↑ [28,40], ↔ [54,71]
*n*-6/*n*-3	↔ [40,72]		↔ [60]	↔ [28,49,60], ↓ [40], ↑ [54]

↑, significantly higher; ↓, significantly lower; ↔, not significantly different. Abbreviations: CE, cholesteryl ester; MUFA, monounsaturated fatty acid; *n*-3 PUFA, omega-3 polyunsaturated fatty acid; *n*-6 PUFA, omega-6 polyunsaturated fatty acid; PL, phospholipid; PUFA, polyunsaturated fatty acid; SFA, saturated fatty acid; TAG, triacylglycerol. Fatty acid abbreviations: 14:0, myristic acid; 16:0, palmitic acid; 16:1*n*-7, palmitoleic acid; 18:0, stearic acid; 18:1*n*-9, oleic acid; 18:2*n*-6, linoleic acid; 18:3*n*-3, α-linolenic acid; 20:4*n*-6, arachidonic acid; 20:5*n*-3, eicosapentaenoic acid; 22:4*n*-6, adrenic acid; 22:5*n*-3, docosapentaenoic acid; 22:6*n*-3, docosahexaenoic acid.

**Table 4:** Studies with blood fatty acid status predicting clinical endpoints.

Author, Year	Country	*n*	Dialysis Vintage (month)	Follow Up (year)	Blood Fraction	FA of Interest	Endpoints (Events) ^†^
Friedman, 2008 [26]	USA	93	N/A	2.1 *	Erythrocyte	*n*-3 index	HR (95% CI) of death: Omega-3 index (below median, 4.69%): 2.48 (0.88–6.95), *p* = 0.085
Hamazaki, 2011 [45]	Japan	176	92.4	5	Erythrocyte	DHA	HR (95% CI) for all-cause mortality: T3 (>8.1%) vs. T1 (<7.2%): 0.43 (0.21–0.88)
Huang, 2012 [47]	Sweden	222	12	1.5	PL	LA, ALA, MA LC *n*-3	HR (95% CI) for all-cause mortality: LA: 0.89 (0.79–0.99) ALA: 0.89 (0.65–1.23) MA: 1.33 (1.17–1.52) LC *n*-3: 0.91 (0.72–1.16)
Friedman, 2013 [23]	USA	400	N/A	1	Total serum, PL	Total SFA, DPA	OR (95% CI) for sudden cardiac death: Total serum Total SFA: 1.01 (1.00–1.02), *p* = 0.0258 DPA: 0.70 (0.51–0.97), *p* = 0.0334 PL DPA: 0.82 (0.69–0.98) ^‡^, *p* = 0.0254
Friedman, 2013 [24]	USA	400	N/A	1	PL	LC *n*-3	OR (95% CI) for sudden cardiac death: Q4 (4.15–15.11%) vs. Q1 (1.27–3.07%): 0.20 (0.08–0.51), *p* = 0.001
Shoji, 2013 [63]	Japan	517	110.4	5	Total serum	(EPA + DHA)/AA ratio	HR (95% CI) for CV events: Q1 (0.63–0.83) vs. Q4 (1.54–2.03): 1.92 (1.25–2.95), *p* = 0.005
Terashima, 2014 [25]	Japan	176	92.4	10	Erythrocyte	DHA, OA	HR (95% CI) for all-cause mortality: DHA T3 (>8.1%) vs. T1 (<7.2%): 0.52 (0.30–0.91) OA T3 (>13.8%) vs. T1 (<13.3%): 0.46 (0.25–0.84)

* median, ^†^ adjusted model for clinical endpoint analyses, ^‡^ data corrected based on personal communication with Friedman et al. [23]. Abbreviations: AA, arachidonic acid; ALA, α-linolenic acid; CI, confidence interval; CV, cardiovascular; DHA, docosahexaenoic acid; DPA, docosapentaenoic acid; EPA, eicosapentaenoic acid; FA, fatty acid; HD, hemodialysis; HR, hazard ratio; LA, linoleic acid; LC *n*-3, long chain *n*-3 PUFAs (the sum of EPA, DPA, and DHA); MA, mead acid; N/A, not available; OA, oleic acid; OR, odds ratio; PD, peritoneal dialysis; PL, phospholipid; Q1, quartile 1; Q4, quartile 4; SFA, saturated fatty acid, T1, tertile 1; T3, tertile 3.

## Supplementary Materials

The following are available online at http://www.mdpi.com/2072-6643/10/10/1353/s1, Table S1: Study selection based on inclusion criteria after reviewing full text, Table S2: Quality assessment of articles reporting clinical endpoints, Table S3: Comparison of FA status of dialysis patients to healthy controls based on dialysis vintage.
