# Supplementary material for: Blood Fatty Acid Status and Clinical Outcomes in Dialysis Patients: A Systematic Review
Source: Nutrients. 2018 Sep 21;10(10):1353. doi: 10.3390/nu10101353 (PMC6213187; doi:10.3390/nu10101353)
Supplement: Supplementary file 1 [file nutrients-10-01353-s001.pdf]

Table S1: Study selection based on inclusion criteria after reviewing full text.

First author, year	Journal	Tittle	Include?	Reason of Exclusion
Ahmad, 1989	Kidney International Supplement	Fatty acid abnormalities in hemodialysis patients: effect of L-carnitine administration.	No	Duplicate publication
An, 2009	Journal of Renal Nutrition	Comparison of fatty acid contents of erythrocyte membrane in hemodialysis and peritoneal dialysis patients.	Yes	
An, 2011	Clinical Nephrology	Association of adiponectin and leptin with serum lipids and erythrocyte omega-3 and omega-6 fatty acids in dialysis patients.	Yes	
An, 2012	Nutrition Research	Omega-3 fatty acid supplementation increases 1, 25-dihydroxyvitamin D and fetuin-A levels in dialysis patients.	Yes	
An, 2012	Prostaglandins, Leukotrienes and Essential Fatty Acids	Effect of omega-3 fatty acids on the modification of erythrocyte membrane fatty acid content including oleic acid in peritoneal dialysis patients.	Yes	
Allawi, 2017	Journal of Pharmaceutical Sciences and Research	Effects of Omega-3 on Vitamin D Activation in Iraqi Patients with Chronic Kidney Disease Treated by Maintenance Hemodialysis.	No	No FA data
Asemi, 2016	Molecular Nutrition & Food Research	Effect of the omega‐3 fatty acid plus vitamin E supplementation on subjective global assessment score, glucose metabolism, and lipid concentrations in chronic hemodialysis patients.	No	No FA data
Asemi, 2016	International Urology and Nephrology	Effects of omega-3 fatty acid plus alpha-tocopherol supplementation on malnutrition–inflammation score, biomarkers of inflammation and oxidative stress in chronic hemodialysis patients.	No	No FA data
Barden, 2015	Journal of Hypertension	n-3 fatty acids reduce plasma 20-hydroxyeicosatetraenoic acid and blood pressure in patients with chronic kidney disease.	No	Non-dialysis
Barden, 2016	Nutrients	n-3 fatty acid supplementation and leukocyte telomere length in patients with chronic kidney disease.	No	Non-dialysis
Bartel, 1982	Metabolism-Clinical and Experimental	Effect of dialysis on serum carnitine, free fatty acids, and triglyceride levels in man and the rat.	No	No FA data
Beavers, 2008	Nephrology	Omega-3 fatty acid supplementation and total homocysteine levels in end‐stage renal disease patients.	No	No FA data
Begum, 2004	Journal of Renal Nutrition	Supplementation with n-3 and n-6 polyunsaturated fatty acids: effects on lipoxygenase activity and clinical symptoms of pruritus in hemodialysis patients.	Yes	
Bowden, 2007	Journal of Renal Nutrition	Effects of omega-3 fatty acid supplementation on vascular access thrombosis in polytetrafluorethylene grafts.	No	No FA data
Bowden, 2009	Journal of Renal Nutrition	Effects of omega-3 fatty acid supplementation on lipid levels in end stage renal disease patients.	No	No FA data
Bowden, 2009	Nutrition in Clinical Practice	Fish oil supplementation lowers C‐reactive protein levels independent of triglyceride reduction in patients with end‐stage renal disease.	No	No FA data
Cacciagiú, 2014	Metabolic Syndrome and	Do insulin resistance conditions further impair the lipid and inflammatory profile in end-	No	No FA data

	Related Disorders	stage renal disease patients on hemodialysis?		
Chapkin, 1983	Translational Research	Characterization of the individual phospholipids and their fatty acids in serum and high-density lipoprotein of the renal patient on long-term maintenance hemodialysis.	No	No FA of interest
Dahwa, 2014	Biomarkers	Variability of oxidative stress biomarkers in hemodialysis patients.	No	No FA data
Dasgupta, 1990	Clinical Physiology and Biochemistry	Abnormal fatty acid profile in chronic hemodialysis patients: possible deficiency of essential fatty acids.	Yes	
Dashti-Khavidaki, 2014	American Journal of Therapeutics	Effects of omega-3 fatty acids on depression and quality of life in maintenance hemodialysis patients.	No	No FA data
Daud, 2012	Vascular Health and Risk Management	Effects of protein and omega-3 supplementation, provided during regular dialysis sessions, on nutritional and inflammatory indices in hemodialysis patients.	No	No FA data
Deger, 2016	Clinical Journal of the American Society of Nephrology	High dose omega-3 fatty acid administration and skeletal muscle protein turnover in maintenance hemodialysis patients.	No	No FA data
Delarue, 2008	British Journal of Nutrition	Fish oil attenuates adrenergic overactivity without altering glucose metabolism during an oral glucose load in haemodialysis patients.	Yes	
Delmas-Beauvieux, 1995	Nephron	Evaluation of red blood cell lipoperoxidation in hemodialysed patients during erythropoietin therapy supplemented or not with iron.	Yes	
Dessì, 2014	Lipids in Health and Disease	Plasma and erythrocyte membrane phospholipids and fatty acids in Italian general population and hemodialysis patients.	Yes	
de Fijter, 1995	Haematologica	Does additional treatment with fish oil mitigate the side effects of recombinant human erythropoietin in dialysis patients?.	Yes	
de Gomez Dumm, 2001	Medicina-Buenos Aires	Lipid abnormalities in chronic renal failure patients undergoing hemodialysis.	Yes	
de Mattos, 2017	Journal of Renal Nutrition	Omega-3 fatty acid supplementation is associated with oxidative stress and dyslipidemia, but does not contribute to better lipid and oxidative status on hemodialysis patients.	Yes	
De Smet, 2001	Clinical chemistry	Heparin-induced release of protein-bound solutes during hemodialysis is an in vitro artifact.	No	No FA of interest
Dhingra, 2016	Environmental Research	Perfluorooctanoic acid and chronic kidney disease: Longitudinal analysis of a Mid-Ohio Valley community.	No	Non-dialysis
Dolegowska, 2007	Renal Failure	Effect of hemodialysis on the content of fatty acids in monolayers of erythrocyte membranes in patients with chronic renal failure.	No	No FA of interest
Eloot, 2013	PLoS One	Does the adequacy parameter kt/vurea reflect uremic toxin concentrations in hemodialysis patients?	No	No FA data
Esaki, 2017	Journal of Renal Nutrition	Effects of a novel nutritional formula enriched with eicosapentaenoic acid and docosahexaenoic acid specially developed for tube-fed hemodialysis patients.	Yes	
Friedman, 2006	American Journal of Kidney Diseases	Fish consumption and omega-3 fatty acid status and determinants in long-term hemodialysis.	Yes	

Friedman, 2008	Journal of Renal Nutrition	Feasibility study of erythrocyte long-chain omega-3 polyunsaturated fatty acid content and mortality risk in hemodialysis patients.	Yes	
Friedman, 2008	Journal of Renal Nutrition	Acute rise of omega-3 polyunsaturated fatty acids during hemodialysis treatment.	No	Duplicate publication
Friedman, 2012	American Journal of Nephrology	Low Blood Levels of Long-Chain n–3 Polyunsaturated Fatty Acids in US Hemodialysis Patients: Clinical Implications.	Yes	
Friedman, 2013	Kidney International	Inverse relationship between long-chain n-3 fatty acids and risk of sudden cardiac death in patients starting hemodialysis.	Yes	
Friedman, 2013	American Journal of Nephrology	Fatty acids and other risk factors for sudden cardiac death in patients starting hemodialysis.	Yes	
Friedman, 2016	Nutrition Research	Association between plasma endocannabinoids and appetite in hemodialysis patients: A pilot study.	Yes	
Ghanei, 2012	Iranian Red Crescent Medical Journal	Efficacy of omega-3 fatty acids supplementation in treatment of uremic pruritus in hemodialysis patients: a double-blind randomized controlled trial.	No	No FA data
Gault, 1992	Nephron	Heparin, fatty acids and sodium, potassium-ATPase inhibition by plasma factors during hemodialysis.	No	No FA of interest
Gharekhani, 2014	Journal of Renal Nutrition	Effects of oral supplementation with omega-3 fatty acids on nutritional state and inflammatory markers in maintenance hemodialysis patients.	No	No FA data
Gharekhani, 2014	DARU Journal of Pharmaceutical Sciences	Potential effects of omega-3 fatty acids on anemia and inflammatory markers in maintenance hemodialysis patients.	No	No FA data
Gharekhani, 2014	European Journal of Clinical Pharmacology	The effect of omega-3 fatty acids on depressive symptoms and inflammatory markers in maintenance hemodialysis patients: a randomized, placebo-controlled clinical trial.	No	No FA data
Gillett, 2004	Saudi Medical Journal	Plasma concentrations of non-esterified fatty acids in chronic renal failure in the United Arab Emirates.	No	No FA data
Girelli, 1992	Clinica Chimica Acta	Red blood cells and platelet membrane fatty acids in non-dialyzed and dialyzed uremies.	Yes	
Harving, 2015	Clinical Nephrology	n-3 polyunsaturated fatty acids and adiponectin in patients with end-stage renal disease.	No	Duplicate publication
Hamazaki, 1984	Kidney International	Effects of fish oil rich in eicosapentaenoic acid on serum lipid in hyperlipidemic hemodialysis patients.	Yes	
Hamazaki, 2009	Clinical Nephrology	The relationship between n-3 long-chain polyunsaturated fatty acids and pulse wave velocity in diabetic and non-diabetic patients under long-term hemodialysis. A horizontal study.	No	Duplicate publication
Hamazaki, 2011	American journal of Nephrology	Docosahexaenoic acid is an independent predictor of all-cause mortality in hemodialysis patients.	Yes	
Hashimoto, 1996	Clinica Chimica Acta	Lipid abnormalities of erythrocyte membranes in hemodialysis patients with chronic renal failure.	No	No FA data
Holler, 199	Peritoneal Dialysis International	Eicosanoid precursors: potential factors for atherogenesis in diabetic CAPD patients?	Yes	
Horiuchi, 1987	European Journal of Clinical	Plasma free fatty acids and protein binding of disopyramide during haemodialysis.	No	No FA data

	Pharmacology			
Hsu, 2010	Nephron Clinical Practice	N-acetylcysteine for the management of anemia and oxidative stress in hemodialysis patients.	No	No FA data
Huang, 2012	Nephrology Dialysis Transplantation	Essential polyunsaturated fatty acids, inflammation and mortality in dialysis patients.	Yes	
Huang, 2013	Journal of Internal Medicine	Clinical determinants and mortality predictability of stearoyl-Co A desaturase-1 activity indices in dialysis patients.	Yes	
Ichimura, 2016	Yakugaku Zasshi	Correlations between Plasma Levels of Anionic Uremic Toxins and Clinical Parameters in Hemodialysis Patients	No	No FA data
Irish, 2017	JAMA Internal Medicine	Effect of fish oil supplementation and aspirin use on arteriovenous fistula failure in patients requiring hemodialysis: a randomized clinical trial.	No	No FA data
Kajbaf, 2016	Journal of Research in Pharmacy Practice	Does Omega-3 supplementation decrease carotid intima-media thickening in hemodialysis patients?.	No	No FA data
Khosroshahi, 2013	Iranian Journal of Kidney Diseases	Effect of omega-3 supplementation on serum level of homocysteine in hemodialysis patients.	No	No FA data
Kim, 2004	Prostaglandins, Leukotrienes and Essential Fatty Acids	Alteration of plasma total F2-isoprostanes before and after hemodialysis in end-stage renal disease patients.	No	No FA of interest
Kim, 2013	Iranian Journal of Kidney Diseases	Comparison of fetuin-A, vitamin D, monounsaturated fatty acid, and vascular calcification on plain radiography between dialysis modalities.	Yes	
Kirkegaard, 2012	British Journal of Nutrition	Marine n-3 fatty acids, atrial fibrillation and QT interval in haemodialysis patients.	No	Duplicate publication
Koorts, 2002	Prostaglandins, Leukotrienes and Essential Fatty Acids	Red blood cell fatty acid profile of chronic renal failure patients receiving maintenance haemodialysis treatment.	Yes	
Kooshki, 2011	Annals of Nutrition and Metabolism	Effects of marine omega-3 fatty acids on serum systemic and vascular inflammation markers and oxidative stress in hemodialysis patients.	No	No FA data
Kooshki, 2011	Renal Failure	Effects of omega-3 fatty acids on serum lipids, lipoprotein (a), and hematologic factors in hemodialysis patients.	No	No FA data
Kuwamura, 2018	Journal of Renal Nutrition	Altered Serum n-6 Polyunsaturated Fatty Acid Profile and Risks of Mortality and Cardiovascular Events in a Cohort of Hemodialysis Patients.	No	Duplicate publication
Lee, 2015	International Journal of Endocrinology	Effect of omega-3 fatty acid on the fatty acid content of the erythrocyte membrane and proteinuria in patients with diabetic nephropathy.	No	Non-dialysis
Lee, 2015	Marine Drugs	The effects of omega-3 Fatty Acid on vitamin d activation in hemodialysis patients: a pilot study.	Yes	
Lucchi, 1993	Nephron	Conjugated diene fatty acids in patients with chronic renal failure: evidence of increased lipid peroxidation?	No	No FA of interest
Lucchi, 2000	Kidney international	Changes in conjugated linoleic acid and its metabolites in patients with chronic renal failure.	No	No FA of interest
Lucchi, 2005	Artificial Organs	Changes in conjugated linoleic acid and palmitoleic acid are correlated to retinol levels in	No	No FA of interest

		chronic renal failure in both hemodialysis and conservative treatment patients.		
Madsen, 2010	Journal of Parenteral and Enteral Nutrition	Effect of Intravenous ω-3 Fatty Acid Infusion and Hemodialysis on Fatty Acid Composition of Free Fatty Acids and Phospholipids in Patients With End-Stage Renal Disease.	No	Duplicate publication
Madsen, 2011	Journal of Renal Nutrition	Marine n-3 polyunsaturated fatty acids in patients with end-stage renal failure and in subjects without kidney disease: a comparative study.	Yes	
Maher, 1998	Nephrology Dialysis Transplantation	Neutropenia and plasma free radical reaction products during haemodialysis.	No	No FA of interest
Marangoni, 1992	Artificial Organs	Plasma lipids and fatty acid levels in chronically uremic patients undergoing blood purification with different methods.	Yes	
Mas, 2016	Clinical Nutrition	A randomized controlled trial of the effects of n-3 fatty acids on resolvins in chronic kidney disease.	No	Non-dialysis
Maeda, 1989	Nephron	Effects of L-carnitine administration on short-chain fatty acid (acetic acid) and long-chain fatty acid metabolism during hemodialysis.	No	No FA data
Mingardi, 1988	The International Journal of Artificial Organs	Factors affecting plasma free fatty acids rise during hemodialysis.	No	No FA data
Mirfatahi, 2016	Iranian Journal of Kidney Diseases	Effects of flaxseed oil on serum lipids and lipoproteins in hemodialysis patients: a randomized controlled trial.	No	No FA data
Moeinzadeh, 2016	Iranian Journal of Kidney Diseases	Effects of omega-3 fatty acid supplementation on serum biomarkers, inflammatory agents, and quality of life of patients on hemodialysis.	No	No FA data
Naini, 2015	Journal of Research in Pharmacy Practice	Effect of Omega-3 fatty acids on blood pressure and serum lipids in continuous ambulatory peritoneal dialysis patients.	No	No FA data
Nakata, 2015	European Journal of Echocardiography	Sudden death prediction by C-reactive protein, electrocardiographic findings, and myocardial fatty acid uptake in haemodialysis patients: analysis of a multicentre prospective cohort sub-study.	No	No FA data
Nakamura, 2008	Clinical and Experimental Hypertension	Renoprotective effect of telmisartan in patients with chronic kidney disease.	No	Non-dialysis
Nakamura, 2008	In Vivo	Serum lipid profile and plasma fatty acid composition in hemodialysis patients-comparison with chronic kidney disease patients.	Yes	
Nishimura, 2014	Nephron Clinical Practice	Clinical potential of oral nicorandil to improve myocardial fatty acid metabolism after percutaneous coronary intervention in hemodialysis patients.	No	No FA data
Oh, 2012	Transplantation Proceedings	Comparison of erythrocyte membrane fatty acid contents in renal transplant recipients and dialysis patients. In	Yes	
Pazda, 2017	Biomedical Chromatography	Suitability of selected chromatographic columns for analysis of fatty acids in dialyzed patients.	Yes	
Peck, 1996	The American Journal of Clinical Nutrition	Effect of three sources of long-chain fatty acids on the plasma fatty acid profile, plasma prostaglandin E2 concentrations, and pruritus symptoms in hemodialysis patients.	Yes	

Perunicic-Pekovic, 2007	Nephrology	Effect of n - 3 fatty acids on nutritional status and inflammatory markers in haemodialysis patients.,	Yes	
Peuchant, 1988	Clinica Chimica Acta	Increase of erythrocyte resistance to hemolysis and modification of membrane lipids induced by hemodialysis.	Yes	
Peuchant, 1994	Free Radical Biology and Medicine	Lipoperoxidation in plasma and red blood cells of patients undergoing haemodialysis: vitamins A, E, and iron status.	Yes	
Poulia, 2011	Journal of Renal Nutrition	Omega-3 fatty acids supplementation does not affect serum lipids in chronic hemodialysis patients.	No	No FA data
Puchades, 2013	Clinical Nephrology	Study of oxidative stress in patients with advanced renal disease and undergoing either hemodialysis or peritoneal dialysis.	No	No FA data
Rasmussen, 2010	Nutrition Research	The content of docosahexaenoic acid in serum phospholipid is inversely correlated with plasma homocysteine levels in patients with end-stage renal disease.	No	Duplicate publication
Ristić, 2006	Renal Failure	Plasma and erythrocyte phospholipid fatty acids composition in Serbian hemodialyzed patients.	Yes	
Ristic-Medic, 2014	The Scientific World Journal	Effects of dietary milled seed mixture on fatty acid status and inflammatory markers in patients on hemodialysis.	Yes	
Rivara, 2015	Journal of Renal Nutrition	A pilot randomized crossover trial assessing the safety and short-term effects of pomegranate supplementation in hemodialysis patients.	No	No FA data
Rivara, 2015	BMC Nephrology	Association of plasma F2-isoprostanes and isofurans concentrations with erythropoiesis-stimulating agent resistance in maintenance hemodialysis patients.	No	No FA data
Roob, 2001	Kidney International	Ex vivo low-density lipoprotein oxidizability and in vivo lipid peroxidation in patients on CAPD.	No	No FA of interest
Rroji, 2016	Journal of Nephrology	Association of advanced age with concentrations of uraemic toxins in CKD.	No	No FA data
Saifullah, 2007	Nephrology Dialysis Transplantation	Oral fish oil supplementation raises blood omega-3 levels and lowers C-reactive protein in haemodialysis patients—a pilot study.	No	Duplicate publication
Sagara, 2016	Cardiovascular Diabetology	Impact of teneligliptin on oxidative stress and endothelial function in type 2 diabetes patients with chronic kidney disease: a case–control study.	No	Non-dialysis
Salahudeen, 2001	Kidney International	Increase in plasma esterified F2-isoprostanes following intravenous iron infusion in patients on hemodialysis.	No	No FA data
Sasagawa, 1998	Journal of Nutritional Science and Vitaminology	The significance of plasma lysophospholipids in patients with renal failure on hemodialysis.	No	No FA of interest
Scheppach, 1988	Clinical Nephrology	Effects of acetate during regular hemodialysis.	No	No FA data
Sertoglu, 2014	Chemistry and Physics of Lipids	Comparison of plasma and erythrocyte membrane fatty acid compositions in patients with end-stage renal disease and type 2 diabetes mellitus.	Yes	
Shearer, 2012	Journal of Renal Nutrition	Plasma fatty acids in chronic kidney disease: nervonic acid predicts mortality.	No	Non-dialysis
Shoji, 2013	American Journal of Kidney Diseases	Serum n-3 and n-6 polyunsaturated fatty acid profile as an independent predictor of cardiovascular events in hemodialysis patients.	Yes	

Sikorska-Wiśniewska, 2017	Renal failure	Disorders of serum omega-3 fatty acid composition in dialyzed patients, and their associations with fat mass.	Yes	
Sørensen, 2015	Journal of Renal Nutrition	The effect of n-3 fatty acids on small dense low-density lipoproteins in patients with end-stage renal disease: a randomized placebo-controlled intervention study.	No	Duplicate publication
Son, 2012	Journal of Renal Nutrition	Association between vascular calcification scores on plain radiographs and fatty acid contents of erythrocyte membrane in hemodialysis patients.	Yes	
Suchitra, 2011	Saudi Journal of Kidney Diseases and Transplantation	The effect of L-carnitine supplementation on lipid parameters, inflammatory and nutritional markers in maintenance hemodialysis patients.	No	No FA data
Svensson, 2006	Clinical journal of the American Society of Nephrology	N-3 fatty acids as secondary prevention against cardiovascular events in patients who undergo chronic hemodialysis: a randomized, placebo-controlled intervention trial.	Yes	
Svensson, 2008	Nephrology Dialysis Transplantation	The effect of n-3 fatty acids on lipids and lipoproteins in patients treated with chronic haemodialysis: a randomized placebo-controlled intervention study.	No	Duplicate publication
Szklarek-Kubicka, 2009	Journal of Renal Nutrition	Effect of intradialytic intravenous administration of ω-3 fatty acids on nutritional status and inflammatory response in hemodialysis patients: a pilot study.	No	No FA data
Taccone-Gallucci, 1986	American Journal of Nephrology	Red blood cell membrane lipid peroxidation in continuous ambulatory peritoneal dialysis patients.	No	No FA data
Taccone-Gallucci, 1989	The International Journal of Artificial Organs	Lack of oxidative damage in serum polyunsaturated fatty acids before and after dialysis in chronic uremic patients.	Yes	
Takamura, 1997	Clinical Chemistry	Effects of uremic toxins and fatty acids on serum protein binding of furosemide: possible mechanism of the binding defect in uremia.	No	No FA data
Talwalker, 1980	The Journal of Clinical Endocrinology & Metabolism	Different mechanisms for the increased enzymatic activity of renin in plasma of patients with chronic renal failure and patients receiving glucocorticoid therapy.	Yes	
Terashima, 2014	Hemodialysis International	Inverse association between docosahexaenoic acid and mortality in patients on hemodialysis during over 10 years.	Yes	
Tikanoja, 1989	Clinica Chimica Acta	Association between increased concentrations of free thyroxine and unsaturated free fatty acids in non-thyroidal illnesses: role of albumin.	No	Duplicate publication
Tsujimoto, 2013	Toxins	Effects of decreased vitamin D and accumulated uremic toxin on human CYP3A4 activity in patients with end-stage renal disease.	No	No FA data
Tsuzuki, 2000	Clinica Chimica Acta	Analysis of 7-ketocholesterol in low density lipoprotein and fatty acid composition in erythrocyte membranes of patients on maintenance hemodialysis and healthy controls.	Yes	
Umemoto, 2016	Atherosclerosis	Reverse association of omega-3/omega-6 polyunsaturated fatty acids ratios with carotid atherosclerosis in patients on hemodialysis.	Yes	
Varga, 1997	Nephron	Relative abundance of some free fatty acids in plasma of uremic patients: Relationship between fatty acids, lipid parameters, and diseases.	No	No FA of interest
Viecelli, 2016	Nephrology	Baseline characteristics of the omega‐3 fatty acids (F ish oils) and A spirin in V ascular access OU tcomes in RE nal D isease (FAVOURED) study.	No	No FA data

Wang, 2016	Journal of Proteome Research	Plasma lipidomics investigation of hemodialysis effects by using liquid chromatography–mass spectrometry.	No	No FA data
Watkins, 2016	The Journal of Nutritional Biochemistry	Serum phospholipid fraction of polyunsaturated fatty acids is the preferred indicator for nutrition and health status in hemodialysis patients.	No	Duplicate publication
Wessel-Aas, 1984	Acta Medica Scandinavica	Hemodialysis and cell toxicity in vitro related to plasma triglycerides, post - heparin lipolytic activity and free fatty acids.	No	No FA of interest
Wessel-Aas, 1984	Acta Medica Scandinavica	The effect of systemic heparinization on plasma lipoproteins and toxicity in patients on hemodialysis and continuous ambulatory peritoneal dialysis.	No	No FA of interest
Westhuyzen, 2003	Annals of Clinical & Laboratory Science	Oxidative stress and erythrocyte integrity in end-stage renal failure patients hemodialysed using a vitamin E-modified membrane.	Yes	
Wong, 2016	Nutrition	Ratio of dietary ω-3 and ω-6 fatty acids—independent determinants of muscle mass—in hemodialysis patients with diabetes.	No	No FA data
Yamamoto, 2015	Scientific Reports	Continuous reduction of protein-bound uraemic toxins with improved oxidative stress by using the oral charcoal adsorbent AST-120 in haemodialysis patients.	No	No FA data
Yerlikaya, 2011	Renal Failure	Plasma fatty acid composition in continuous ambulatory peritoneal dialysis patients: an increased omega-6/omega-3 ratio and deficiency of essential fatty acids.	Yes	
Yoshimoto-Furuie, 1999	Nephron	Effects of oral supplementation with evening primrose oil for six weeks on plasma essential fatty acids and uremic skin symptoms in hemodialysis patients.	Yes	
Zabel, 2010	Journal of Renal Nutrition	Adherence to fish oil intervention in patients with chronic kidney disease.	No	Duplicate publication
Zabel, 2010	Journal of Human Nutrition and Dietetics	, R., Ash, S., King, N., Naslund, E., & Bauer, J. (2010). Gender differences in the effect of fish oil on appetite, inflammation and nutritional status in haemodialysis patients.	Yes	
Zakaria, 2017	International Urology and Nephrology	The impact of fish oil and wheat germ oil combination on mineral-bone and inflammatory markers in maintenance hemodialysis patients: a randomized, double-blind, placebo-controlled clinical trial.	No	No FA data

Abbreviation: FA, fatty acid.

Table S2: Quality assessment of studies reporting clinical endpoints.

Study	Q1	Q2	Q3	Q4	Q5 (a)	Q5 (b)	Q6 (a)	Q6 (b)	Q9	Q10	Q11	Q12
Friedman, 2008 [26]	Yes	Can't tell	Yes	Yes	Yes	Yes	Yes	Yes	Can't tell	Can't tell	No	Can't tell
Hamazaki, 2011 [45]	Yes	No	Yes	Yes	Yes	Yes	Yes	Yes	Yes	Can't tell	Yes	Yes
Huang, 2012 [47]	Yes	Yes	Yes	Yes	Yes	Yes	Yes	Yes	Can't tell	Yes	No	Can't tell
Friedman, 2013 [23]	Yes	Yes	Yes	Yes	Yes	Yes	Yes	Yes	Can't tell	Yes	Yes	Yes

Friedman, 2013 [24]	Yes	Yes	Yes	Yes	Yes	Yes	Yes	Yes	Yes	Yes	Yes	Yes
Shoji, 2013 [63]	Yes	Yes	Yes	Yes	Yes	Yes	Yes	Yes	Yes	Yes	Yes	Yes
Terashima, 2014 [25]	Yes	No	Yes	Yes	Yes	Yes	Yes	Yes	Yes	Can't tell	Yes	Yes

Footnote: Question 7 and 8 are open-ended questions and excluded from the quality assessment.

Abbreviation: Q, question.

Table S3. Comparison of FA status of dialysis patients to healthy controls based on dialysis vintage.

	Total serum/plasma		PL		Erythrocyte	
	DV <72 months	DV ≥72 months	DV <72 months	DV ≥72 months	DV <72 months	DV ≥72 months
SFA						
14:0		↔[18,59]			↔[49,54], ↓[28]	↔[58]
16:0	↑[34]	↔[18,59]		↔[60]	↔[28,49,54],	↔[58,60]
18:0	↔[34]	↔[18,, ↓[59]		↔[60]	↔[28,49], ↓[54]	↔[58,60]
Total SFA				↔[60]	↔[28,49], ↓[54]	↔[60], ↑[69]
MUFA						
16:1*n*-7	↑[34]	↔[18]		↔[60]	↔[49,54], ↓[28]	↑[60]
18:1*n*-9	↑[34,64]	↑[18,59]		↑[60]	↑[28,49,54]	↔[58,60]
Total MUFA				↑[60]	↑[49,54], ↓[28]	↔[69]
n-6 PUFA						
18:2*n*-6		↓[18,59]	↓[38]	↔[42,60]	↓[38,49], ↑[28,54]	↓[69], ↔[58,60]
20:4*n*-6	↓[34,64],↑ [51]	↓[18,59]	↓[38]	↔[60], ↑[42]	↔[49,54], ↑[28], ↓[38]	↔[58,60], ↑[29]
22:4*n*-6	↓[34]	↔[59]		↔[60]	↔[49], ↓[28]	↔[58,60], ↓[69]
Total *n*-6 PUFA				↓[60]	↑[28,54], ↓[49],	↑[29], ↓[60]
n-3 PUFA						
18:3*n*-3	↓[64]	↔[18]	↔[38]		↓[28,38], ↔[49], ↑[54]	↓[29]
20:5*n*-3	↓[34,51,64]		↓[38]	↓[42,60]	↔[28,38], ↓[49,54]	↔[29,58], ↓[60]
22:5*n*-3	↓[34]	↔[18,59]		↔[60]	↔[28,,49,54],	↔[29,60], ↓[69]
22:6*n*-3	↓[34,51,64]	↔[18,59]	↓[38]	↓[60], ↔[42]	↓[38,54], ↔[28,49]	↓[58,60], ↔[29,69]
n-3 Index			↔[38]		↔[28,38], ↓[54]	
Total *n*-3 PUFA	↓[64]			↓[60]	↔[28,49], ↓[54]	↔[29], ↓[60]
PUFA						
Total PUFA					↓[49], ↑[28], ↔[54]	↓[69]
n-6/*n*-3				↔[60]	↔[28,49], ↑[54]	↔[60]

↑, significantly higher; ↓, significantly lower; ↔, not significantly different. Abbreviation: CE, cholesteryl ester; DV, dialysis vintage; MUFA, monounsaturated fatty acid; *n*-3 PUFA, omega-3 polyunsaturated fatty acid; *n*-6 PUFA, omega-6 polyunsaturated fatty acid; PL, phospholipid; PUFA, polyunsaturated fatty acid; SFA, saturated fatty acid; TAG, triacylglycerol. Fatty acid abbreviation: 14:0, myristic acid; 16:0, palmitic acid; 16:1*n*-7, palmitoleic acid; 18:0, stearic acid; 18:1*n*-9, oleic acid; 18:2*n*-6, linoleic acid; 18:3*n*-3, α-linolenic acid; 20:4*n*-6, arachidonic acid; 20:5*n*-3, eicosapentaenoic acid; 22:4*n*-6, adrenic acid; 22:5*n*-3, docosapentaenoic acid; 22:6*n*-3, docosahexaenoic acid.
